# Asthma diagnosis and treatment – 1016. Is atopy in people aged 40 and over related to fixed airflow obstruction?

**DOI:** 10.1186/1939-4551-6-S1-P16

**Published:** 2013-04-23

**Authors:** Michael J Abramson, Brett G Toelle, Alan L James, Richard Wood-Baker, Deborah Burton, Wei Xuan, David P Johns, A Sonia Buist, Guy B Marks

**Affiliations:** 1 Epidemiology & Preventive Medicine, Monash University, Australia; 2 Woolcock Institute of Medical Research, Sydney, Australia; 3 Sir Charles Gairdner Hospital, Perth, Australia; 4 Royal Hobart Hospital, Hobart, Australia; 5 Charles Sturt University, Orange, Australia; 6 Menzies Research Institute, Hobart, Australia; 7 Oregon Health & Science University, Portland, OR, USA

## Background

The Tucson and Normative Ageing studies showed atopy was not related to airflow obstruction in later life, when known asthmatics were excluded. However these studies were based on pre-bronchodilator spirometry.

## Aim

To examine independent associations between atopy and post-bronchodilator airflow obstruction in people aged 40 years and over.

## Methods

We used data from the BOLD-Australia study. Persons aged 40 years and over were randomly selected from the electoral roll, either directly or via a two stage sampling procedure, in centres based in four Australian States. Spirometry was performed before and after administration of salbutamol 200 μg, according to a standardised protocol with careful attention to quality control. Subjects with post-bronchodilator FEV_1_/FVC ratio < 0.7 were classified as GOLD stage 1 or higher. Subjects who also had FEV_1_ < 80% predicted were classified as GOLD stage 2 or higher. Subjects who had a post-bronchodilator increase in FEV_1_ > 12% of pre-bronchodilator value and > 200 ml were classified as “reversible”. Atopy was assessed by skin prick tests (SPTs) to house dust mites (*D. pteronyssinus* and *D. farinae*), cat, dog, cockroach, *Alternaria*, *Aspergillus*, rye grass and mixed grass pollens. Subjects with any allergen SPT ≥ 4 mm were classified as atopic.

## Results

Post-bronchodilator spirometry and SPT data were available for 2,767 subjects (51.4% female, 12.1% aged ≥ 75). The prevalence of GOLD stage 1 or higher was 15.9%, the prevalence of GOLD stage 2 or higher was 6.8%, and 4.7% had bronchodilator reversibility. One in nine subjects self-reported current asthma and 50.5% had ever been smokers. The prevalence of atopy in this population was 42.7%.

The associations between atopy and airflow obstruction did not differ by self-reported asthma or smoking status (all interactions p > 0.2). However atopy was associated with GOLD stage 2 after adjustment for sex, self-reported current asthma and smoking status (OR=1.56, 95%CI 1.13-2.17). As expected, atopy was related to the presence of bronchodilator reversibility (OR=1.89, 95%CI 1.32-2.72).

## Conclusions

The apparent association of atopy with fixed airflow obstruction using post-bronchodilator spirometry in people aged 40 years and over may be due to clinical heterogeneity.

